# Maturity Levels of Public Safety Applications using Unmanned Aerial Systems: a Review

**DOI:** 10.1007/s10846-021-01462-7

**Published:** 2021-08-23

**Authors:** Merlin Stampa, Andreas Sutorma, Uwe Jahn, Jörg Thiem, Carsten Wolff, Christof Röhrig

**Affiliations:** 1 Institute for the Digital Transformation of Application and Living Domains (IDiAL), Dortmund University of Applied Sciences and Arts, Otto-Hahn-Str. 23, 44227 Dortmund, Germany; 2 Faculty of Information Technology, Dortmund University of Applied Sciences and Arts, Sonnenstr. 96, 44139 Dortmund, Germany

**Keywords:** Unmanned aerial systems, Unmanned aerial vehicles, Public safety, Disaster, Emergency

## Abstract

Unmanned Aerial Systems (UAS) are becoming increasingly popular in the public safety sector. While some applications have so far only been envisioned, others are regularly performed in real-life scenarios. Many more fall in between and are actively investigated by research and commercial communities alike. This study reviews the maturity levels, or “market-readiness”, of public safety applications for UAS. As individual assessments of all applications suggested in the literature are infeasible due to their sheer number, we propose a novel set of application categories: Remote Sensing, Mapping, Monitoring, Human-drone Interaction, Flying Ad-hoc Networks, Transportation, and Counter UAV Systems. Each category’s maturity is assessed through a literature review of contained applications, using the metric of Application Readiness Levels (ARLs). Relevant aspects such as the environmental complexity and available mission time of addressed scenarios are taken into account. Following the analysis, we infer that improvements in autonomy and software reliability are the most promising research areas for increasing the usefulness and acceptance of UAS in the public safety domain.

## 1 Introduction

Protecting the public from threats is a complex and labor-intensive endeavor. The scale of threats can vary wildly, from comparably small ones affecting a single person or only material goods to large-scale disasters—"a sudden, calamitous event that seriously disrupts the functioning of a community or society and causes human, material, and economic or environmental losses that exceed the community's or society's ability to cope using its resources" [1]. Every threat can be addressed in one or more of four stages [1]:

1. *Mitigation:* Reducing the risk of a threat occurring.
2. *Preparedness:* Developing a response plan.
3. *Response:* The actions taken immediately after the threat occurred.
4. *Recovery:* Efforts to return to normalcy.

The advantages of Unmanned Aerial Systems (UAS) for a broad range of missions in various stages have long been identified and are actively investigated, as the rest of this paper will show. A UAS consists of one or multiple Unmanned Aerial Vehicles (UAVs, commonly referred to as *drones*) and associated elements such as ground control stations, localization systems, and more. Initially developed for military purposes, they are nowadays popular with hobbyists and professionals from various industries alike [2, 3]. UAVs[1] constitute popular research subjects for

[1]Throughout the rest of this article, we use the terms "UAV" and "drone" interchangeably to refer to UAVs for civil use—excluding military models.

✉ Merlin Stampa
merlin.stampa@fh-dortmund.de

Andreas Sutorma
andreas.sutorma@fh-dortmund.de

Uwe Jahn
uwe.jahn@fh-dortmund.de

Jörg Thiem
joerg.thiem@fh-dortmund.de

Carsten Wolff
carsten.wolff@fh-dortmund.de

Christof Röhrig
christof.roehrig@fh-dortmund.de

1 Institute for the Digital Transformation of Application and Living Domains (IDiAL), Dortmund University of Applied Sciences and Arts, Otto-Hahn-Str. 23, 44227 Dortmund, Germany

2 Faculty of Information Technology, Dortmund University of Applied Sciences and Arts, Sonnenstr. 96, 44139 Dortmund, Germany roboticists since they can not only replace human labor in dull, dirty, or dangerous tasks, like many ground-based robots. They can also enable applications infeasible for humans due to their ability to fly or even hover, with spatial dimensions and operational costs that are often magnitudes smaller than those of crewed aircraft.

A growing number of public safety authorities regularly employ UAS for their work (Fig. 1). However, UAS have not reached their full potential in this sector yet and their adoption can even be met with skepticism from practitioners [4]. The impediments partially lie in regulatory restrictions, acquisition costs, and other organizational issues—but also the fact that not all UAS-based solutions have reached a maturity that meets the challenging requirements of many scenarios. In comparison to the commercial domain, public safety applications (especially during the response stage) are more frequently characterized by the need for briefest response times in complex, initially unknown, and dynamically changing environments.

With plummeting prices and advancements in the hardware and software of UAS in recent years, the scientific community was able to accelerate its efforts tremendously. Through searches for the term `UAS` (and closely related terms[2]) on the academic search engine Google Scholar[3], we discovered a steep increase in the number of published articles per year (Fig. 2), indicating the growing relevance of this field. The search engine reported a total of ca. 749,000 results as of January 2021. A similar trend can be observed in the subset concerned with UAS in the public safety context, with currently over 378,000 results in total.

While the majority of these studies naturally focus on the scientific advancement of specific UAS-related technologies, the main goal of the review at hand is to investigate how *mature* public safety applications of UAS already are. Or in other words, how far have UAS applications moved out of the hands of researchers and into the ones of practitioners? We put a focus on the work of fire departments, since our ongoing collaboration with the *Institute of Fire Service and Rescue Technology Dortmund (IFR)*[4] has particularly sensitized us to the perspective of firefighters and rescue teams.

**Outline** In Section 2, we present our method for finding, selecting, and evaluating references. This method is first applied in Section 3 to assess existing reviews of UAS in public safety applications. Section 4 briefly reviews the metric of *Application Readiness Levels* used for the subsequent maturity ratings. Derived from existing categorizations in the literature, we propose a novel categorization scheme in Section 5, which also contains said maturity assessments. Section 6 discusses our findings, while Section 7 concludes the study.

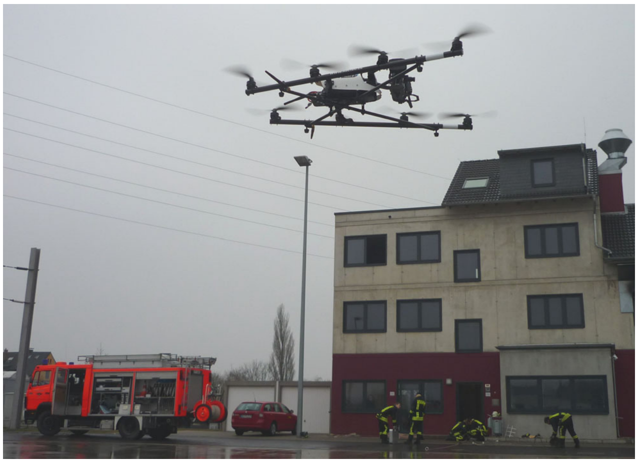

**Fig. 1** UAV flown during a fire drill in Dortmund. Photo by courtesy of the *Institute of Fire Service and Rescue Technology, Dortmund*

## 2 Methodology

The proposal of a taxonomy for UAS in public safety applications and assessments of their maturity levels are complex due to the lack of existing systematics and structure in this field. The relevant sources and data are quite broad and not yet sufficiently structured and analyzed. Therefore, we believe that our contribution should serve as a first step into building the required structure and theory in order to assess the maturity of UAS in public safety applications. This first step has to be taken despite the risk of some shortcomings regarding completeness and validation. In that sense, our contribution is a proposal for a taxonomy and maturity assessment. Though we do not really develop grounded theory, our approach follows Bowen's proposal to support a grounded theory with an audit trail [5]. The authors decided to describe their methodology and

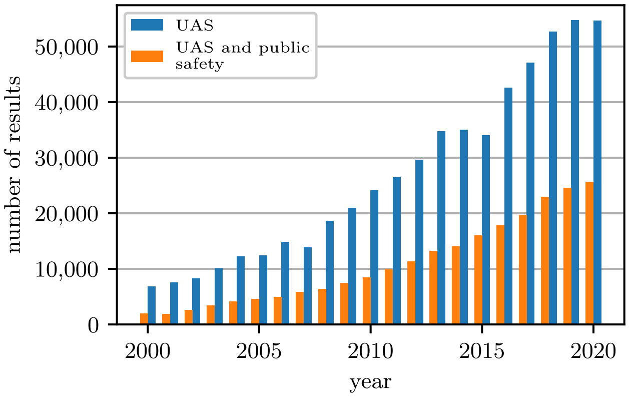

**Fig. 2** The number of search results on Google Scholar for UAS (and related terms), compared to its conjunction with public safety (also with related terms) per year from 2000 to 2020. The numbers were obtained using a script that sequentially issues HTTP requests to the website and parses the returned HTML

[2]Closely related terms of "UAS" and "public safety" are listed in Appendix A.

[3]https://scholar.google.com/

[4]http://feuerwehr.dortmund.de/

decisions in a detailed audit trail to make it repeatable and allow improvements where the methodology lacks rigor and validity. For some steps, we had to take pragmatic decisions in order to move forward. These decisions are documented, and the steps can be revisited with more detailed research and more rigor.

Our method to find, select, and evaluate sources for Sections 3 and 5 leans on *Systematic Literature Reviews* (SLRs) [6]. As academic sources, we used the online database IEEE Xplore[5] and open access articles from various publishers found in the databases of arXiv[6] and Google Scholar. Furthermore, we used articles found and archived during previous, UAS-related works of ours. Occasionally we searched for non-scientific publications on Google[7] to enhance our assessments. All search queries used for this study are presented in the Appendix A. A found publication had to satisfy all of the following inclusion criteria to be selected for further examination:

- The publication addresses UAS in its title or abstract using any relevant term or description.
- It discusses at least one UAS application in the context of public safety.
- It contains explicit information about this application's maturity. For example, it describes performed test flights, demonstrations, or successful real-life missions.
- It must contain at least a descriptive concept of the application and not only preliminary technology research.
- It was published in 2010 or later. We suspected more recent publications are more likely to exhibit higher maturity levels.

As Fig. 2 illustrated, the number of publications concerned with UAS in the public safety context is vast and seems to increase steadily. If—despite our best efforts to create sufficiently targeted queries—an academic database search returned more than 100 results, we sorted the list by *relevance* (how closely the results matched the query, as determined by the search engine itself) and used only the first 100 results for further processing. This procedure was especially required for Google Scholar, which a) presented more than 10,000 results for a majority of the queries and b) does not provide an option for sorting other than relevance. To save time in the later steps, we preselected the results obtained here by their titles.

## 3 Meta-review of UAS in Public Safety Applications

Previous studies reviewed public safety applications of UAS. Next to the inclusion criteria listed in Section 2, found studies had to meet the criterion of actually being (or at least containing) a review on UAS applications. This criterion was added because the search term "survey" also yielded articles using other meanings of the word (as in "land survey").

### 3.1 Comprehensive Reviews

UAVs as Mobile Sensing Platforms (MSPs) in the domains *smart cities & public safety* as well as *civil security & disaster response* were reviewed in [7]. The survey lists numerous case studies for various applications, leading to the conclusion that "such mobile sensing / actuation platforms have already matured to the point where they are widely considered as a viable addition to existing applications and approaches" [7]. The group stated that the emergence of UAV swarms is inevitable, and the "usefulness of such swarms will dramatically increase with growing autonomy". This is similar to conclusions found in a review about optimization approaches for civil UAV applications: "future research should work out *dynamic planning schemes*" for a range of relevant drone operations [and] develop *approaches to deal with data uncertainty* [8].

In [9] the author presented a survey on proposed and implemented drone applications in Africa, where many local governments actively support the technology. Examples include delivering healthcare-related goods, wildlife monitoring, and even predicting atrocities in conflict-ridden regions. The study lists the benefits and challenges of drones before naming considerations for proper and safe drone usage, including community participation, consistent regulatory frameworks, and more.

### 3.2 UAS for Disaster Management

Several reviews specifically focused on the *disaster response* domain. Reference [10] lists case studies of UAVs used for imagery collection following hurricanes, typhoons, and earthquakes. The authors concluded that UAVs are viable data collection tools for such events. Reference [11] presented a systematic literature review of UAVs in humanitarian relief applications, charting this research field's rapid growth through a statistical analysis. Out of 117 surveyed papers, 62 addressed the recovery stage of "natural sudden-onset" disasters. Furthermore, they showed that most surveyed publications focused on improving the equipment's performance, especially for imagery and mapping applications. A similar study on the use of UAS in humanitarian relief was conducted in [12]. The authors summarized that while the use of UAS is more widespread in the military, humanitarian aid projects are mostly still in the concept and testing phase. UAS developments and concepts in this area take place

[5] https://ieeexplore.ieee.org/
[6] https://arxiv.org
[7] https://www.google.com in the use cases mapping (e.g., of flood risks), deliveries (e.g., medical products), search and rescue (e.g., after natural catastrophes), monitoring changes (e.g., buildings destroyed after earthquakes), public health prevention (e.g., combating mosquitoes plagues), agriculture (e.g., monitoring crops), monitoring climate change (e.g., monitor glaciers with thermal cameras), demining (e.g., searching for shifted mines after floods) and protecting civilians and peacekeeping (e.g., detect hidden troops) [12].

State of the art in UAV-based photogrammetry[8] (especially for earthquakes, volcanic activity, and landslides) was studied in [13]. The authors concluded that this technique has proven useful in various scenarios and will continue to gain traction. Reference [14] examined the combination of UAVs and Wireless Sensor Networks (WSNs) for disaster management, highlighting open issues in network stability. The authors analyzed 29 case studies in terms of the addressed disaster stage, used technology (WSN or UAV), and performed application (monitoring, information fusion, situational awareness, damage assessment, standalone communication system, search and rescue) [14, Tab. 1]. Worth mentioning is furthermore [15, Sec. 2.G], which briefly lists UAV usage examples from disasters such as the 2011 Fukushima Daiichi nuclear disaster or the 2013 earthquake in Port au Prince, Haiti.

### 3.3 UAS for Forest Fire Monitoring

Three of the selected reviews focused on *forest fire monitoring*. The authors of [16] reviewed the development of UAV-based forest firefighting systems and present an overview of vision-based fire detection techniques. Reference [17] states that this application is an active field of research with numerous experiments already performed, but with plenty of room for improvement before it can be considered sufficiently mature—especially regarding fire detection algorithms, integration, and testing under realistic conditions. The authors of [18] emphasized increased autonomy as a key enabler for this use case.

### 3.4 Other Reviews

A recent survey on drone usage in public safety agencies was performed by members of the U.S. Department of Commerce's National Institute of Standards and Technology [19]. In line with the research group's focus, it stressed the need for improved wireless communication during missions and gathered requirements for future, UAV-based solutions. Additionally, the survey "identified a potential gap in the use of drones in some public safety operations where drones might improve [...] operational performance, but it also showed that agencies are making attempts to add UAS expertise and skilled drone pilots" [19].

A sociological study on institutional realities and public perceptions of police use of UAVs in Canada was presented in [20]. The authors examined service flight logs and concluded that most operational flights were "dedicated to tasks associated with assisting persons in immediate risk (e.g., missing persons) or gathering evidence for an identified crime" [20].

Multiple countries employed UAS in attempts to combat the COVID-19 pandemic. A collection of related use cases, exemplifying how versatile UAS can be, was presented in [21]: crowd surveillance, public announcements, temperature screenings, spraying disinfectants, and the delivery of medical supplies.

### 3.5 Contribution of this Study

The listed reviews provide insight into the state of the art in various applications of UAS from a dominantly technological point of view. In contrast, the review at hand aims to develop a more in-depth analysis of the maturity levels, or "market-readiness", of UAS-based public safety applications. To the best of our knowledge, no previous study has attempted a comparable investigation in this domain.

## 4 Application Readiness Levels

Comparing the maturity levels of various applications requires a consistent metric. For this purpose, we decided to employ the nine-step *Application Readiness Level* (ARL) index conceived by NASA to track and manage a project's progression [22, 23]. ARLs are an adaptation of the well-known *Technology Readiness Levels* (TRLs) [24] for assessing technology development and risk. The main reason we chose ARLs over TRLs was to emphasize our focus on the maturity of *applications*, which can depend on a variety of technologies, rather than the UAS technology on its own. Another small advantage of ARLs for this study is the more explicit consideration of the end user's perspective, e.g., including the need for documentation and training. However, ARLs and TRLs are sufficiently similar to each other that they can be used almost interchangeably. In the following, we briefly recite the ARL concept to improve the readability of the later sections. Figure 3 presents an overview.

ARLs can be divided into three major phases:

Phase I (ARL 1–3): Discovery and feasibility.
Phase II (ARL 4–6): Development, testing, and validation.

[8] Generating measurements or maps from photographs.

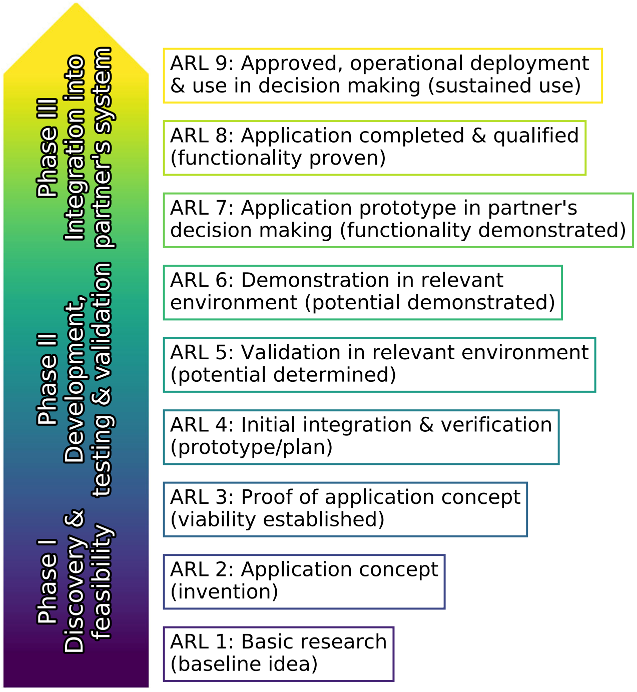

**Fig. 3** Overview of *Application Readiness Levels*. Adapted from [22, p. 2] to fit this article's layout

Phase III (ARL 7–9): Integration into partner's system.

Many research projects end before or in the development phase (II), whereas the final integration and fine-tuning of the system (phase III) is often left to companies or the end users. The individual levels can be summarized as follows:

ARL 1: Basic scientific concepts and insights are observed and reported, providing the foundation for application ideas.

ARL 2: The formulation of the application concept and the individual components has begun. The full system is still speculative.

ARL 3: Feasibility studies are conducted, providing a proof-of-concept. The components have been tested and validated independently.

ARL 4: Basic components are integrated into a prototype system to verify that they will work together.

ARL 5: The prototype is integrated with reasonably realistic supporting elements. The potential advantages of the new application are clearly articulated.

ARL 6: The prototype and its advantages have been demonstrated in a relevant or simulated operational environment.

ARL 7: The prototype and all pre-deployed components are fully integrated, tested, and demonstrated in the end-user's operational environment to win the partner's confidence.

ARL 8: The application, in its final form, is proven to work under expected conditions. The user documentation is complete.

ARL 9: This application system has seen successful and sustained use in the operational environment.

Generally, ARLs are not meant to measure the performance of individual tools or models—they are used to quantify their readiness for use in a decision-making process. There still can be significant room for improvement after reaching ARL 9, e.g., increased performance or lower costs.

## 5 Application Categories and their Maturity Levels

As previously stated in Section 2, the vast number of UAS applications renders individual examinations infeasible. However, many applications show substantial similarities, allowing one to sort them into a manageable list of *application categories*. For example, tasks to transport material are mostly similar from a UAV's perspective regardless of this material being vaccines, humanitarian aid, fire extinguishing agents, or something else entirely. Several categorizations have been proposed or re-utilized in the literature [7, 11, 14, 20]. Although good starting points, we found these categorizations inadequate for our review because they include areas outside of the public safety domain (e.g., precision agriculture) or miss application categories that we deem relevant (e.g., intercepting rogue drones). We have therefore derived a novel categorization, primarily based on the respectively required capabilities and hardware components. The categories are namely *Remote Sensing, Mapping, Monitoring, Human-drone Interaction (HDI), Flying Ad-Hoc Networks (FANETs), Transportation* and *Counter UAV Systems (C-UAS)*. An overview of these categories, including exemplary technical and mission goals, is presented in Table 1. A proper validation is pending, but we have confidence in this proposal since it a) overlaps with the existing categorizations and b) could successfully be applied to all references we found for the subsequent maturity assessments.

Each of the following subsections describes one application category and presents this category's maturity assessment. The assessments themselves arose from the following process:

1. We performed a literature search (see Section 2) for publications addressing applications of the category[9]. Naturally, we included findings from the existing reviews (Section 3).

[9]The exact queries and other details are listed in the Appendix A.

**Table 1** Overview of UAS application categories used in this study

| Application category | Technical goal | Exemplary mission goal | Examples of required hardware and capabilities |
|---|---|---|---|
| Remote Sensing | Gather image or other sensor data. | Gain an aerial overview, find missing persons. | Cameras or other sensors. |
| Mapping | Reconstruct a digital map of an area. | Assess structural damages. | Same as *Remote Sensing* plus data fusion. |
| Monitoring | Continually monitor an area over a long-term period. | Detect developing forest fires. | Same as *Remote Sensing* plus increased autonomy, automatic recharging, pattern recognition. |
| Human-drone Interaction | Communicate with people in the UAV's vicinity. | Guide civilians towards evacuation routes. | Human-Machine Interfaces, e.g., displays, speakers, or microphones. |
| Flying Ad-Hoc Networks | Create a radio network between UAS and users on the ground. | Provide cellphone service to civilians in need. | Antennas, routers, automated recharging. |
| Transportation | Deliver cargo. | Provide humanitarian aid to people in hard-to-access areas. | Attachable container, possibly pick-up and release or dispersion mechanism. |
| Counter UAV Systems | Stop rogue drones from flying. | Ensure the safety of starting or landing crewed aircraft at airports. | Radar, net cannons, signal jammers. |

2. In each article, we investigated which ARL characteristics (Section 4) were fulfilled. For example, an article describing a successful demonstration in an operational environment was said to reach ARL 6. A definite assignment to an ARL was omitted if it became evident that previously assessed publications in the same category scored higher.
3. The maturity of a category is the maximum ARL reached by one of the publications selected for it. Further investigations for the category were omitted if a publication reached the highest possible ARL 9.

Not necessarily all the articles selected by us for particular categories are referenced, but only those which directly influenced our assessments.

**Limitations** Our ARL ratings of the proposed application categories (and the individual publications within) are potentially biased and should be treated with due caution. Despite our best efforts to achieve scientific rigor and objectivity, a certain degree of uncertainty and bias cannot be ruled out for a number of reasons, e.g.:

– Our assessments are limited by the literature we discovered.
– The authors of investigated publications (especially in the context of commercial or preliminary applications) may have overstated positive and understated (or even omitted) negative results.
– The assessments are of qualitative nature and as such also prone to personal biases.

Nonetheless, we find the overall picture emerging from the entirety of all assessments to be coherent and have confidence in the conclusions we have drawn from it (Sections 6 and 7).[10]

## 5.1 Remote Sensing

**Description** Many UAS applications are purely concerned with data acquisition and require the UAVs to carry not much more than one or more sensors. We group these applications into the *Remote Sensing* category, also identified by the term *Imagery* (if no sensors other than cameras are required). Variants of this category include *Situational Awareness* (Fig. 4), *Searching* (e.g., for missing persons), and *Inspection* (e.g., to assess damages). In our opinion, these variants are mostly discerned by the chosen flight paths and required level of detail in the data but are otherwise equivalent.

[10]We would like to invite inclined readers to discuss the assessments, and point to relevant publications we missed, by writing an email to the authors.

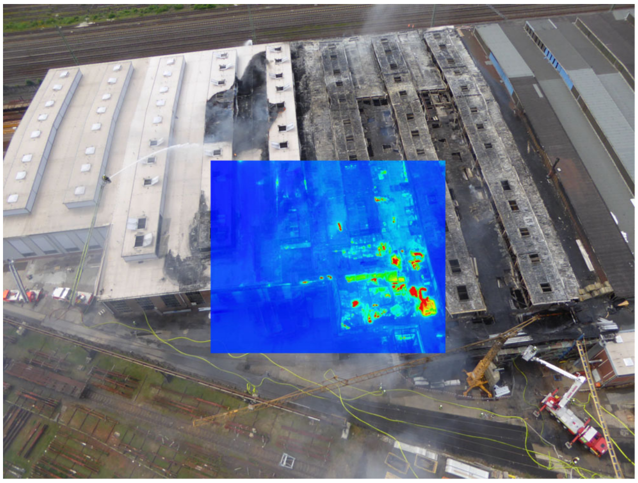

**Fig. 4** Drone view of a major fire in Dortmund overlayed with infrared measurements. The drone was used to track the fire's progression. Found via [28], photo by courtesy of the *Dortmund Fire Department*

**Assessment** This category is arguably the most mature, as numerous public safety authorities routinely use UAS for this purpose nowadays. Supplementary to examples found in existing reviews [10, 15, 20, 21], we would like to highlight the following news articles:

- Tracking the progression of flames and finding optimal positions for fire hoses during the 2019 fire of Notre-Dame de Paris [25].
- Searching for missing persons [26].
- Assessing a criminal suspect's weaponry [27].

The *Remote Sensing* or *Imagery* category has matured to a point where periodicals for professionals such as the German *Feuerwehr-Magazin* [Fire Department Magazine] have published special issues about UAS for firefighters and similar professions, including overviews of commercially available models and legal considerations [28]. The sustained use clearly warrants a 9 on the ARL scale.

## 5.2 Mapping

If the remotely gathered data is processed and fused to create a digital model of the environment, we assign the application to the *Mapping* category. The created maps may represent the physical structure of the environment but could also model other types of data, such as temperatures or concentrations of harmful substances [29]. *Mapping* can be seen as an extension of *Remote Sensing*—there is often no distinction made in the literature. Nevertheless, due to the increased requirements on processing capabilities and the high number of publications explicitly addressing this topic, we decided that a designated category is justified. The difficulty of *Mapping* applications strongly depends on the available mission time and environmental complexity. Therefore, we have furthermore decided to split *Mapping* into two subcategories.

### 5.2.1 Assessment Mapping

**Description** On one side of the spectrum, there are scenarios where a map of a medium-sized area (e.g., a single building and its immediate surroundings) is desired, with plenty of time available for data acquisition and processing. Applications fitting this description fall into the *Assessment Mapping* subcategory, which can also be seen as a map-based expansion of *Inspection*.

**Assessment** Numerous articles have shown the viability of UAV-based *Assessment Mapping* in a variety of scenarios, such as geological disasters (earthquakes, volcanic activity, and landslides) [13, 15, 30, 31], nuclear disasters [15], fire risk estimation [32], traffic accident reconstruction [33], or crime scene investigation [34]. Most publications in this area focus on post-disaster assessment [11], but case studies addressing the mitigation and preparation stages exist as well [32, 35]. A recent review on established and emerging technologies for structural damage mapping showed the substantial evolution this field has seen in the last decades [36]. The quality of maps produced in the aftermath of earthquakes can allow civil engineers to estimate the risk of building collapse [31]. Most often, the UAVs are flown manually or along pre-defined paths [36]. Nowadays, there are multiple professional photogrammetry software solutions readily available for this purpose [37]. Consequential to the sustained use, we determined the overall ARL for *Assessment Mapping* applications to be 9.

### 5.2.2 Emergency Mapping

**Description** On the other side, a requirements workshop we conducted with members of the *Institute of Fire Service and Rescue Technology Dortmund* pointed to the desire for mapping capabilities during the response stage of large-scale disasters, such as fires in industrial complexes or flash floods [38]. This application area is referred to as *Emergency Mapping*, the "creation of maps, geo-information products and spatial analyses dedicated to providing situational awareness emergency management and immediate crisis information for response" [39] or sometimes *Real-Time Mapping* [40]. Such scenarios can necessitate the usage of a swarm of UAVs utilizing a distributed system architecture [41, 42] since a single-vehicle system might not be able to cover the area fast or detailed enough. At the same time, the number of available human pilots is typically limited. Hence, the UAS would have to operate autonomously. I.e., the UAVs need to navigate in complex terrain (e.g., with numerous physical

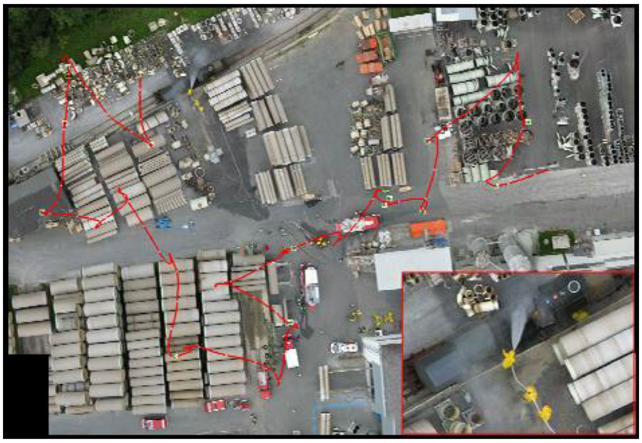

**Fig. 5** Overview image computed from a set of 40 pictures [43, Fig. 3]. Reprinted from Proceedings for the International ISCRAM Conference (43), [43], Fast aerial image acquisition and mosaicking for emergency response operations by collaborative UAVs, pp 1-5, with the author's permission obstacles and sensor disturbances stemming from fire or smoke) while not disturbing the core response efforts or imposing too much cognitive load on the already-stressed operators.

**Assessment** The most advanced system capable of *Emergency Mapping* we discovered is the one presented in [43]. The authors used three autonomous UAVs during a fire drill to generate a regularly updated 2D overview image (Fig. 5). Their close collaboration with the firefighters on site lead us to assign ARL 7 to their publication.

For even more complex scenarios, involving 3D or even mixed indoor/outdoor mapping, there has been—to the best of our knowledge—no work that presented a viable, fully integrated system. However, we would like to mention that there are comprehensive research projects that have been or are currently pushing the boundaries of robot-based emergency mapping in terms of scale and complexity, such as INACHUS (focusing on the localization of entrapped victims) [40], TRADR (persistent environment and action models as well as human-robot teaming) [44], Eins3D (instantaneous 3D mapping) [45], A-DRZ[11] (integration and usability) [46], or our own, Software4Robots (software development processes) [38, 47]. We assigned a maximum of ARL 3 to publications stemming from said research projects, but ARL 4 might be reached soon.

To summarize, we find the maturity level of *Emergency Mapping* application to strongly depend on the complexity of targeted scenarios. While high maturity levels have been reached for 2D mapping from an unobstructed bird's-eye view (ARL 7 for [43]), 3D mapping applications in more demanding environments have just begun to move into phase II (development, testing, and validation).

[11]The authors are partially involved in the A-DRZ joint research project.

### 5.3 Monitoring

**Description** Next to *Mapping*, we consider continuous, long-term *Monitoring* (also identified by the term *Surveillance*) to be another extension of *Remote Sensing*. Continuous coverage of an area enables the detection of irregularities such as developing fires or unauthorized intruders (e.g., via machine learning techniques). Since the long mission times render the employment of human pilots impractical, a cost-efficient solution to a *Monitoring* problem requires a substantial degree of autonomy and robustness [17, 18] through capabilities such as automated recharging, fault tolerance, self-diagnosis, and more.

**Assessment** As recent reviews have pointed out, there has been sizable research on forest fire monitoring [17, 18]. Relevant technologies (e.g., fire detection or spread prediction) have seen promising progress. However, many still need to be tested under more realistic conditions and integrated into a verifiable system [17]. This limits our rating of this application's maturity to ARL 3. An example can be found in the *iWBB* project [48, 49], which proposed to employ UAS as an addition to conventional early detection systems for forest fires. The approach relies on stationary visual sensors to produce the earliest alarms for developing fires, which are then automatically verified by a dispatched multicopter equipped with additional sensors. After a fire is extinguished, an unmanned blimp can hover over the area for a longer time to act as a so-called "fire guard" and detect possible re-ignitions. The team validated most of their essential components in valuable outdoor tests but did not perform an integration.

On a much smaller scale than forests, there are companies such as *Sunflower Labs Inc.* [50] working on UAS-based home surveillance systems. Their approach is quite similar to the iWBB proposal: automated UAVs are sent out to investigate alarms produced by stationary, ultrasonic sensors. When the UAV is not in use, it is securely stored and charged in its base station. At the time of writing, the company has disclosed demonstration videos from a residential house designated for testing purposes—but no detailed reports on performed experiments or customer experiences. Hence, we believe their product currently reaches ARL 6.

The following categories require the UAVs to carry not only sensors, but also actuators to interact with their environment.

### 5.4 Human-drone Interaction (HDI)

**Description** The category *Human-drone Interaction* contains applications that involve direct interactions between a UAV and nearby people, usually through visual or acoustic signals. For example, drone-mounted loudspeakers could be used to issue public announcements to a crowd of people [21]. A drone additionally equipped with a microphone could enable bilateral communication between entrapped civilians and rescue units in emergency scenarios.

**Assessment** In multiple countries, drones equipped with loudspeakers were regularly used to address offenders of curfews issued during the COVID-19 pandemic [21, 51]. This fact justifies a rating of ARL 9.

Other notable applications falling into the *HDI* category are still developing. For example, reference [52] proposed a UAS-based evacuation guidance system and verified the path planning component through a short experiment (ARL 2).

### 5.5 Flying Ad-hoc Networks (FANETs)

**Description** A common problem in the response and recovery stages of disasters is the lack of communication infrastructure (e.g., following the destruction of cell phone towers). UAS could potentially compensate for this issue by creating "Flying Ad-Hoc Networks" (FANETs) between themselves and end-users (Fig. 6). FANETs are an immensely popular field of research, exemplified through a large number of publications and comprehensive surveys, e.g. [53–56].

**Assessment** Virtually all publications we examined focus on package routing protocols, UAV positioning, hardware design or other technological aspects—evaluated through a numerical analysis, simulations, or rather simple flight tests. According to our findings, the *FANET* application category faces the same barrier as large-scale *Monitoring* (Section 5.3): lack of integration and testing under realistic conditions. This circumstance limits our maturity assessment to ARL 3. In terms of tested components, the most advanced publication we were able to discover performed real flight tests with two UAVs and a stationary gateway [57]. The authors investigated the performance of a role changing strategy but did not integrate other essential components of a complete FANET system (e.g., automated flight controls).

Publications addressing the *preparation* phase are slightly harder to find since most authors assume that a stationary network is in place before a disaster occurs. This assumption may not always hold in rural areas. One project that attempted to solve this issue with a so-called *High Altitude Platform System* was Facebook's *Aquila* [58]. They claim to have successfully tested individual components such as a designated aircraft or millimeter-wave communication but did not integrate them before the project was stopped in 2018 (again, ARL 3).

### 5.6 Transportation

**Description** The *Transportation* category requires the ability to carry cargo. In its simplest form, this can be done with a manually attachable container to deliver small packages. More complex variants may include automated pick-up & release or dispersion mechanisms.

**Assessment** UAS-based transportation has become increasingly interesting for companies and institutions in the logistics sector. Their endeavors focus on achieving fully autonomous deliveries to make them cost-effective. At the same time, applications from the public safety domain (currently) relying on human pilots have already proven their viability, delivering goods such as

- blood and medical supplies [9],
- disinfectants to spray public infrastructure in areas struck by COVID-19 [21], and

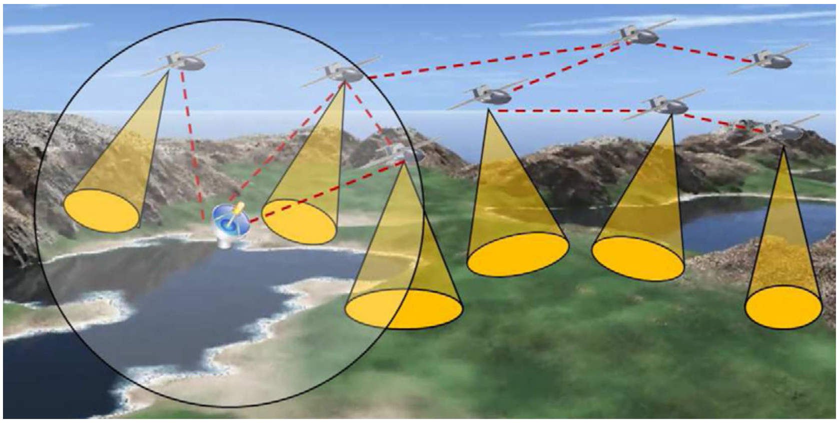

**Fig. 6** A FANET scenario to extend the scalability of multi-UAV systems [53, Fig. 1]. Reprinted from Ad Hoc Networks, Vol 11(3), [53], Flying Ad-Hoc Networks (FANETs): A survey, pp 1254–1270, Copyright (53), with permission from Elsevier.

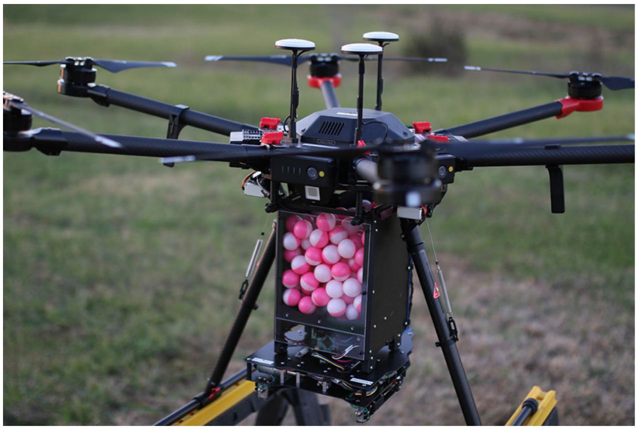

**Fig. 7** A hexacopter equipped with a system to disperse self-igniting balls for starting controlled fires, as reported by [59]. Photo by courtesy of *Drone Amplified Inc*

– self-igniting balls to start controlled burns as a means to contain wildfires [59] (shown in Fig. 7).

The sustained use warrants ARL 9. On a side note, a UAS with transportation capabilities could also be used to collect samples (e.g., from a chemically contaminated ground) for later analysis. To the best of our knowledge, sample collection has so far only been performed in commercial or research contexts [60] but not in the public safety domain, which is why we decided to exclude this aspect from this review.

### 5.7 Counter UAV Systems (C-UAS)

**Description** In most countries, UAVs are subject to regulatory frameworks. They often restrict flying near critical infrastructure (e.g., airports or power plants), over certain heights, or under specific circumstances (e.g., at night or over a crowd of people). Following the growing popularity of consumer-grade quadcopters in recent years, an emerging field is the defense against malfunctioning or illegally flown drones [61]. An effective countermeasure could not only stop rogue UAVs in the air (response). It may also deter perpetrators from ever starting (mitigation) or provide valuable evidence after a successful take-down (recovery).

There are several approaches to tackle this problem; the most dominant are net cannons and radio jammers. While currently available *Counter UAV Systems* (CUS or C-UAS) are dominantly ground-based or hand-held, some solutions employ so-called *hunter* or *interceptor drones* to bring down rogue UAVs (around 9%, according to [61]). UAV-based solutions come with the advantage of increased flexibility and operational range.

**Assessment** There are several reviews on the employed technologies, the current market situation, and future trends [61–63]. In contrast, academic publications on UAS-based C-UAS solutions, detailed enough for maturity assessments, are—according to our findings—almost non-existent. An exception is reference [64], which proposed a navigation, guidance and control architecture to generate interception paths. This component was validated on a real drone intercepting virtual target drones, thus reaching ARL 2.

To better understand the maturity of drone-based C-UAS, we extended our search to non-academic sources. The Tokyo Metropolitan Police Department has set up a drone interceptor squad after an incident in 2015, where a drone carrying trace amounts of radioactive material was found on the Prime Minister's official residence [61]. Their interceptors carry a net under them and are manually flown over the target such that it will get stuck in the net. A demonstration video from a drill exists [65], but we could not find reports proving the squad's effectiveness, let alone real-life captures—limiting our rating to ARL 7 for now. One reason why real-life captures have not been reported may be the complexity of manually piloting a drone for an interception mission. Companies such as Fortem Technologies [66] or Delft Dynamics [67] are currently developing systems that can track, capture, and tow away hostile drones autonomously. Another approach is to destroy the target by simply colliding the interceptor into it [68]. Similar to developers of UAS-based home surveillance systems (Section 5.3), these companies have so far disclosed demonstration videos at best, but no reports on performed tests or customer experiences (ARL 6).

### 5.8 Hybrid Applications

Of course, many applications do not fit solely into one of the presented categories. For example, a UAS for large-scale firefighting and rescue missions can transition from mapping to monitoring after a sufficiently detailed map was created [38, 43]. With regard to our maturity assessments, these hybrid applications do not require special consideration since the contained categories can be examined independently from each other.

## 6 Discussion

Our assessments are graphically summarized in Fig. 8. Applications that can be performed with human pilots and comparably simple hardware (in the categories *Remote Sensing*, *Assessment Mapping*, *Human-drone Interaction* and *Transportation*) exhibit the highest maturity levels. The readiness of other applications generally decreases with the required degrees of autonomy and reliability, aspects

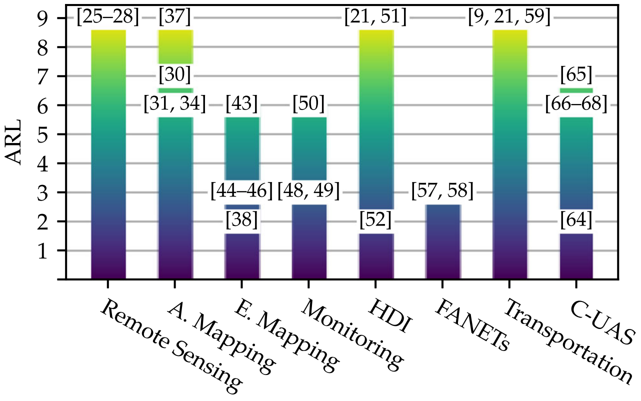

**Fig. 8** A visual summary of our ratings, mapping the application categories to their achieved maturity levels. The annotations contain notable references that contributed to our assessments which the following subsections will discuss in more detail. UAS that can counter rogue drones (*C-UAS*) or perform continuous *Monitoring* of comparably small areas might be the next to see regular use, as there are multiple companies actively developing them. *Emergency Mapping* applications are found on a similar maturity level if the targeted scenario is comparably simple. The currently least mature applications are those requiring UAV swarms rather than a single drone. This is the case for *Flying Ad-hoc Networks (FANETs)* but also *Emergency Mapping* if the targeted scenarios are sufficiently complex.

### 6.1 Autonomy

Autonomy, i.e., a technical system's ability to make its own decisions (e.g., to prevent damages), is a field of interest for researchers. The development of fully autonomous UAS can be compared to the development of Mars rovers, presented in [69]. Sojourner (landed 1997) was the first rover to drive autonomously on another planet, and every successor (Spirit and Opportunity 2004, Curiosity 2012) exhibited gradually increasing levels of autonomy. The development of the Mars rovers shows the possibilities of autonomous systems but still needs to be transferred to UAS with its demands and constraints, some shown in [70]. One approach often used to categorize autonomy levels is the SAE J3016 standard (levels of driving automation) [71]. SAE J3016 describes six SAE categories, from SAE level 0 (no automation) to SAE level 5 (full automation). Other approaches from the field of robotics are compared in [72]. The paper furthermore proposed a taxonomy of 10 levels of robot autonomy for human-robot interaction (HRI), which seems suitable for UAS. In [73], the evolution of a UAV autonomy classification taxonomy is presented. This taxonomy helps to determine the autonomy level of a UAV and where to categorize it within one of the 10 given autonomy control levels (ACLs).

The robotics community has already produced many viable solutions to different aspects of autonomy. For example, a review of path planning techniques was presented in [74]. The authors of [75] compared existing Simultaneous Localization and Mapping (SLAM) algorithms to their solution named RTAB-Map, and mission management and decision-making strategies were surveyed in [76]. Future research will have to integrate such solutions into a full-fledged system and test them in relevant scenarios to achieve higher UAS autonomy levels. While autonomy can be considered mandatory for UAV swarm applications [7], it can also be tremendously beneficial for applications that employ a single UAV. It can boost performance and cost-efficiency [36, Sec. 4.2], reduce risks (allowing safe flights outside of direct visual contact), and reduce the stress imposed on human operators.

### 6.2 Reliability

To be usable for real-life missions, all implemented solutions must exhibit a sufficient degree of reliability—which can be defined as the probability that a system will satisfactorily perform the task for which it was designed or intended under stated conditions for a specified time. While most available hardware platforms can already be seen as sufficiently reliable, more work must be done on the software side to fully benefit from any advances in UAS autonomy. In the past years, tremendous progress has been made in the field of flight controllers (a review was presented in [77]), which abstract from the low-level control of individual rotors to simple commands such as *takeoff* or *go to waypoint*, with some of them using standardized protocols such as MAVLink [78]. To enable the reliable operation of more complex, autonomous behavior, future systems will have to be built using sophisticated design and validation processes, software architectures, and designated toolchains [47].

## 7 Conclusion

This work assessed the maturity levels of UAS applications in the public safety sector through a literature review of both academic and non-academic sources. Grouping the most common UAS applications into a novel set of *application categories* and assessing the applications they contain allowed us to derive a universal insight: the two most promising research fields for the UAS community going forward are autonomy and reliability. Improvements in these aspects would not only increase the performance and efficiency of systems currently flown manually, e.g., for *Remote Sensing*, *Assessment Mapping*, *Human-drone Interaction* or *Transportation* applications. It would also enable applications that are not yet feasible or sufficiently cost-effective with human pilots, such as long-term and large-scale *Monitoring* and *Emergency Mapping*, *Flying Ad-hoc Networks*, or drone-based *Counter UAV Systems*.

The barriers to achieving UAS autonomy are mostly the same faced by ground-based robotics: unreliable sensors, limited processing times, and limited communication bandwidths, to name a few. Some of these problems are even more pressing in UAS due to the need to navigate in 3D rather than 2D space, disturbances by wind, smaller payloads, and more. Similarly, the desire for reliable software is not limited to UAS but rather universal. There are numerous ongoing research endeavors concerned with these challenges. Simultaneously, advancements in self-driving car technology, robot-supported logistics, and military applications continue to benefit the field. At this point, the question at hand seems to be *when* we will see truly autonomous UAS in public safety missions rather than *if*.

## Appendix A: Details of the Literature Searches

*ps_terms* is used to abbreviate:

**Funding** Open Access funding enabled and organized by Projekt DEAL. This research has been funded by the German Federal Ministry of Education and Research in the framework of Software4Robots (BMBF, project number 13FH009IX6).

**Author Contributions** Conceptualization: Merlin Stampa; Project administration: Merlin Stampa; Methodology: Merlin Stampa, Carsten Wolff; Investigation – literature search: Merlin Stampa; Investigation – data analysis: Merlin Stampa, Andreas Sutorma, Uwe Jahn; Visualization: Merlin Stampa; Writing – original draft preparation: Merlin Stampa; Writing – review and editing: Merlin Stampa, Andreas Sutorma, Uwe Jahn, Jörg Thiem, Carsten Wolff, Christof Röhrig; Funding acquisition: Carsten Wolff, Christof Röhrig, Jörg Thiem; Resources: Carsten Wolff, Christof Röhrig, Jörg Thiem; Supervision: Christof Röhrig.

All authors are engaged in the joint research project *Software4Robots*, which provided the framework for this study.

## Declarations

**Conflict of Interests** The authors declare that they have no conflict of interest.

**Open Access** This article is licensed under a Creative Commons Attribution 4.0 International License, which permits use, sharing, adaptation, distribution and reproduction in any medium or format, as long as you give appropriate credit to the original author(s) and the source, provide a link to the Creative Commons licence, and indicate if changes were made. The images or other third party material in this article are included in the article's Creative Commons licence, unless indicated otherwise in a credit line to the material. If material is not included in the article's Creative Commons licence and your intended use is not permitted by statutory regulation or exceeds the permitted use, you will need to obtain permission directly from the copyright holder. To view a copy of this licence, visit http://creativecommons.org/licenses/by/4.0/.

## Appendix A: Details of the Literature Searches

Table 2 lists the search terms used for the literature reviews in this study. Also listed are the numbers of results considered for our assessments. The academic search engines we employed offer varying options to search specific fields of their databases. In Table 2, we use the following markers to denote which fields were searched:

T: Title. On IEEE Xplore this is equivalent to the option “Document Title”, on arXiv to “title”, and to “allintitle” on Google Scholar. A: Abstract or metadata. IEEE Xplore offers “All Metadata”, arXiv “Abstract”. Google Scholar does not provide this option, so it searched for the terms in the entire document.

**Table 2:** Details of the literature searches performed for this study. T: terms searched in the title, A: terms searched in all metadata (IEEE Xplore), abstract (arXiv), or entire document (Google Scholar)

Section	All	Section 3	Section 5.2	Section 5.3	Section 5.5	Section 5.7
Terms	T(*uas_terms)* AND A(*ps_terms)* AND	T(review OR survey)	A(mapping OR photogrammetry)	T(monitoring OR surveillance)	T(FANET OR network OR communication)	A(C-UAS OR CUS OR intercept* OR rogue)
Executed		2021-01-04	2021-01-11	2021-01-15	2021-01-15	2021-01-14
Results considered		164	145	125	171	12

We use *uas_terms* to abbreviate the following query:

UAS OR "unmanned aerial system" ORUAV OR "unmanned aerial vehicle" ORRPAS OR "remotely piloted aircraft system"OR drone*

On Google Scholar, only the shorter terms (UAS, UAV, RPAS, drone⋆) were used in order to stay within the character limit.

*ps_terms* is used to abbreviate:

"public safety" OR "civil security" OR"humanitarian relief" ORdisaster OR emergency ORfire OR rescue OR police

The application categories *Remote Sensing*, *Human-drone* *Interaction*, and *Transportation* (Sections 5.1, 5.4, and 5.6) already reached ARL 9 through the examined reviews (Section 3) and articles we had archived from previous works of ours, so we omitted detailed searches for them.
